# Foreign Correspondence

**Published:** 1890-07

**Authors:** W. H. Williamson

**Affiliations:** Aberdeen, Scotland


### Foreign Correspondence.

To the Editor:

Dear Sir,—In the very practical paper of Dr. Ottolengui, in your journal, the matter of plaster impressions is referred to, coloring the water used for the plaster of the model being recommended. In Kingsley's "Oral Deformities," coloring the water used for either impression or model is spoken of, while in "The American System of Dentistry" the method is not mentioned at all, the reader being told to use either shellac or sandarach varnish, to the use of which, however, your essayist rightly objects, the soap solution being quite sufficient when the method of coloring is adopted. What I have used, and my father before me, for many years, seems to me quite unobjectionable, and fulfils one or two conditions not attained by the method mentioned. It is simplicity itself, and merely consists in the admixture of a grain or two of colored powder, sold by chemists as "rose pink," with the plaster used for the impression. This small quantity gives it a delicate pink color, and the mixture has very much the appearance of strawberry or raspberry ice-cream, perhaps not quite so deep, but, at any rate, it serves in great measure to take away from the mind of the patient the disagreeable idea of having raw, undisguised plaster put in the mouth. The color also serves to distinguish your impression from your model plaster. The mixture can easily be made by your office-boy, and, for that matter, I do not see why dealers should not have all their impression-plaster colored in this way. I do not like the idea of the model being colored, a pure white model being the more acceptable to most dentists, I imagine. Again, in separating the impression from the model, the colored portion is more distinctly appreciated by the eye in breaking away pieces, and, therefore, that portion should be the one forming the impression which lies uppermost in taking it off.

I enclose a tiny sample for color.

Wishing the International all success,

I am yours faithfully,

W. H. Williamson.

Aberdeen, Scotland.

